# Supplementary material for: A Load-Carrier Perspective Method for Evaluating Land Resources Carrying Capacity
Source: Int J Environ Res Public Health. 2022 May 1;19(9):5503. doi: 10.3390/ijerph19095503 (PMC9102171; doi:10.3390/ijerph19095503)
Supplement: Supplementary file 1 [file ijerph-19-05503-s001.zip › ijerph-1674618-supplementary.pdf]

Supplementary information

Supplementary Table S1 The value of indicators $C_{P,1}$ - $C_{P,4}$, $C_{L,1}$ - $C_{L,5}$, and $C_{E,1}$ - $C_{E,2}$ in the 9 county-level administration districts in 2019.

	$C_{P,1}$ (kg)	$C_{P,2}$ (m^3)	$C_{P,3}$ (m^2)	$C_{P,4}$ (m^2)	$C_{L,1}$ (m^2)	$C_{L,2}$ (m^2)	$C_{L,3}$ (m^2)	$C_{L,4}$ (m^2)	$C_{L,5}$ (m^2)	$C_{E,1}$ (ton)	$C_{E,2}$ (kg)	
											TN	TP
Yuzhong	0	5200200	45902	2182989	6060293	3430179	376941	2231079	2390264	28629	14887	6698
Dadukou	853000	23020200	12335972	1766358	14324534	6919780	704416	1192216	1758331	703292	44923	21790
Jiangbei	2456000	51073200	19654870	8655976	27385491	11341215	2082057	3617086	3898046	1654994	94222	47425
Shapingba	10265000	130221000	25577388	11382134	43671184	14765605	2003351	17088099	2674145	3350338	266743	148134
Jiulongpo	12317000	132521400	39690232	13835581	48196961	14022178	2015239	6604618	3874611	3438092	331382	185038
Nan'an	2064000	67527000	16196699	8040428	40579852	13348524	2062094	6252858	4713988	2224069	166808	86851
Beibei	44442000	198703800	30119391	4985453	56686238	10917027	1911188	6285755	5430175	7521427	324557	202489
Yubei	109850000	338218200	49517448	17582051	107907842	42785796	6822563	11075705	10708370	13462766	839007	524247
Ba'nan	215175000	566832600	27550754	12252109	88543196	11994073	1941409	6077559	3991379	21392884	827211	578260

Supplementary Table S2 The value of indicators $L_{P,1}$ - $L_{P,4}$, $L_{L,1}$ - $L_{L,5}$, and $L_{E,1}$ - $L_{E,2}$ in the 9 county-level administration districts in 2019

	$L_{P,1}$ (kg/person)	$L_{P,2}$ (m^3/person)	$L_{P,3}$ (m^2/person)	$L_{P,4}$ (m^2/person)	$L_{L,1}$ (m^2/person)	$L_{L,2}$ (m^2/person)	$L_{L,3}$ (m^2/person)	$L_{L,4}$ (m^2/person)	$L_{L,5}$ (m^2/person)	$L_{E,1}$ (ton/person)	$L_{E,2}$ (kg/person)	
											TN	TP
Yuzhong	421.29	112	111.87	11.59	29.5	10.54	2.83	11.95	10.54	2.5	0.05	0.02
Dadukou	421.29	195	111.87	11.59	36	10.54	2.83	11.95	10.54	8.2	0.32	0.14
Jiangbei	421.29	204	111.87	11.59	30.8	10.54	2.83	11.95	10.54	7.24	0.26	0.11
Shapingba	421.29	181	111.87	11.59	40.5	10.54	2.83	11.95	10.54	9.24	0.43	0.19
Jiulongpo	421.29	189	111.87	11.59	41.7	10.54	2.83	11.95	10.54	7.17	0.41	0.18
Nan'an	421.29	194	111.87	11.59	35.8	10.54	2.83	11.95	10.54	8.13	0.28	0.12
Beibei	421.29	294	111.87	11.59	59.5	10.54	2.83	11.95	10.54	9.27	0.88	0.38
Yubei	421.29	150	111.87	11.59	45	10.54	2.83	11.95	10.54	8.9	0.94	0.40
Ba'nan	421.29	216	111.87	11.59	83.5	10.54	2.83	11.95	10.54	5.86	1.56	0.66

To obtain the value of all indicators shown in **Supplementary Tables S1 and 2**, land use data from the third Chongqing land use survey database should be reclassified by the standard published by National Land and Resources Standardization Techinical Committee [2], as shown in **Supplementary Table S3**.

Supplementary Table S3 Land use classification from the third land use survey database

Classifications	Descriptions
Cultivated land	All areas are used for the production of crops, such as rice, soybean, corn, vegetable, tobacco, and other types of cultivated crops. It refers to code 01 from the third land use survey database
Land for orchards, tea, rubber	These areas are used for perennial woody crops such as orchards, tea, and rubber. It refers to code 02 from the third land use survey database
Arboreal woodland	Arboreal woodland with tree canopy density ≥ 0.2. It refers to code 0301 from the third land use survey database
Bamboo groves	The forest land where bamboo groves grow and the canopy density ≥ 0.2. It refers to code 0302 from the third land use survey database
Shrubby woodland	Shrubby woodland with shrub coverage ≥ 0.4. It refers to code 0305 from the third land use survey database
Other forest land	It refers to code 0307 from the third land use survey database
Grassland	Areas are dominated by herbaceous vegetation. It refers to code 04 from the third land use survey database
Land for commercial services	Areas are mainly used for commerce and service industry. It refers to code 05 from the third land use survey database
Industrial land	Theses land areas are mainly used for industrial production and material storage. It refers to code 06 from the third land use survey database
Residential land	Theses land areas are mainly used for human living and its ancillary facilities. It refers to code 07 from the third land use survey database
Land for public administration and public services	It includes three types of land use, including land for organ groups, the press and publishing, and public facilities. It refers to code 08H1 and 0809 from the third land use survey database
Land for science, education, and culture	It refers to code 08H2 from the third land use survey database
Greenland	It refers to code 0810 from the third land use survey database
Transportation land	Land areas are mainly used for internal travel by transportation means. It refers to code 1002, 1004 and 1005 from the third land

Classifications	Descriptions
	use survey database
Other developed land	It refers to code 05, 1001, 1003, 1006, 1007, 1008 and 1009 from the third land use survey database
Water	All areas of open water, referring to code 11 from the third land use survey database
Other land	It refers to code 12 from the third land use survey database

Supplementary S1 Calculations of indicators measuring for land resources carriers

$C_{P,1}$ is total grain yield which is produced by cultivated land. By referring to Chongqing Statistical Yearbook published in 2020, the value of $C_{P,1}$ in the 9 county-level administration districts in 2019 can be obtained, as shown in **Supplementary Table S1.**

$C_{P,2}$ is the volume of available freshwater resources. To obtain the value of $C_{P,2}$ in the 9 county-level administration districts in 2019, Chongqing Water Resources Bulletin is used for collecting the volume of freshwater resources (Vol) and the proportion of surface freshwater resources (k), and this paper finds that k equals to 1. On the other hand, by referring to A Guide to Water Resources Assessment: SL/T 238-1999, the availability rate of surface freshwater resources (λ) equals to 0.54 which is the same in the 9 county-level administration districts. By applying these data into equations (1)-(3), $C_{P,2}$ can be calculated, as shown in **Supplementary Table S1.**

For the value of indicators $C_{P,3}$-$C_{P,4}$, and $C_{L,1}$-$C_{L,5}$, they can be obtained through ArcGIS (Version 10.7). According to land use classification shown in **Supplementary Table S3**, land use data from the third Chongqing land use survey database can be reclassified, and can be summed in the 9 county-level administration districts. The results are presented in **Supplementary Table S1.**

$C_{E,1}$ is total carbon storage. By applying carbon density (see **Supplementary Table S4**) and land use data into InVEST model (Version 3.9.0),

these spatial data is converted into raster data (resolution: 1m×1m), and the value of indicator C_total_{xyz} can be obtained. And then, by referring to equation (4) through ArcGIS (Version 10.7), $C_{E,1}$ in the 9 county-level administration districts in 2019 are calculated, as shown in **Supplementary Table S1.**

Supplementary Table S4 Carbon density in every land use in Chongqing (unit: ton·ha^{-1})

Classifications	Above-ground carbon density	Below-ground carbon density	Soil organic carbon density	Dead organic carbon density
Cultivated land	1.7	0.32	80.7	0
Land for orchards, tea, rubber	4.55	8.69	82.29	13
Arboreal woodland	48.94	42.35	115.9	13
Bamboo groves	33.21	0.45	0.45	0.59
Shrubby woodland	10.66	25.9	57.95	1.18
Other forest land	5.33	12.95	28.98	0.59
Grassland	17.89	79.59	100.95	3.42
Land for commercial services	0	0	0	0
Industrial land	0	0	0	0
Residential land	0	0	0	0
Land for public administration and public services	0	0	0	0
Land for science, education, and culture	0	0	0	0
Greenland	38.25	5.9	58.55	7.5
Transportation land	0	0	0	0
Other developed land	0	0	0	0
Water	0	0	0	0
Other land	0	0	0	0

$C_{E,2}$ is total volume of TN retention or total volume of TP retention. All spatial data first is converted into raster data (resolution: 1m×1m). By applying DEM data, land use data, percipitation, Biophysical_table (see **Supplementary Table S5**), threshold flow accumulation (valuing 50), borselli k parameter

(valuing 2), subsurface critical length (valuing 800, unit:m), and subsurface maximum retention efficiency (valuing 0.8) into InVEST model (Version 3.9.0), the value of indicator $X_{\exp,xyz}$ can be obtained. And then, by referring to equations (5)-(6) through ArcGIS (Version 10.7), $C_{E,2}$ in the 9 county-level administration districts in 2019 are calculated, as shown in **Supplementary Table S1.**

Supplementary Table S5 Biophysical data in every land use in Chongqing (unit: kg·ha^{-1})

Classifications	The total volume of TN in grid (*x*,*y*) with land use type *z*	The total volume of TP in grid (*x*,*y*) with land use type *z*	Maximum retention efficiency in grid (*x*,*y*) with land use type *z* (%)	Critical length in grid (*x*,*y*) with land use type *z* (m)
Cultivated land	29	12	0.25	25
Land for orchards, tea, rubber	0.15	0.05	0.95	150
Arboreal woodland	0.15	0.05	0.95	150
Bamboo groves	0.15	0.05	0.95	150
Shrubby woodland	0.15	0.05	0.95	150
Other forest land	0.15	0.05	0.95	150
Grassland	0.2	0.09	0.9	100
Land for commercial services	20	9	0.05	10
Industrial land	20	9	0.05	10
Residential land	20	9	0.05	10
Land for public administration and public services	20	9	0.05	10
Land for science, education, and culture	20	9	0.05	10
Greenland	0.15	0.05	0.95	150
Transportation land	20	9	0.05	10
Other developed land	20	9	0.05	10
Water	0.001	0.001	0.05	15

Classifications	The total volume of TN in grid (*x*,*y*) with land use type *z*	The total volume of TP in grid (*x*,*y*) with land use type *z*	Maximum retention efficiency in grid (*x*,*y*) with land use type *z* (%)	Critical length in grid (*x*,*y*) with land use type *z* (m)
Other land	0.2	0.02	0.05	5

Supplementary S2 Calculations of indicators measuring for land resources loads

$L_{P,1}$ is per capita grain consumption, which is the sum of F_1, F_2, F_3, F_4 and F_5. As these data used to calculate F_1, F_2, F_3, F_4 and F_5 are missing in a county-level administration district, this paper collects these data in a municipality-level administration district. First, by referring to measurement models introduced by Tang and Li [1], this paper applies the proportion of urban permanent population (valuing 0.67), the proportion of rural permanent population (valuing 0.33), per capita grain household consumption for eating in urban area (valuing 106.22, unit: kg/person), per capita grain household consumption for eating in rural area (valuing 186.83, unit: kg/person), the proportion of grain non-household consumption in grain household consumption for urban area (valuing 1.64), and the proportion of grain non-household consumption in grain household consumption for rural area (valuing 0.23), into measurement model to obtain the value of F_1, valuing 263.51 kg/person.

Second, by applying total output of liquor (valuing 113400000, unit: kg), total output of beer (valuing 675058500, unit: kg), the grain consumption coefficient of liquor (valuing 2.33), the grain consumption coefficient of beer (valuing 0.15), and the total permanent population (valuing 31243200, unit: person), into measurement model, the value of F_2 can be obtained, valuing

11.70 kg/person.

Third, the value of F_3 is 124.70 kg/person, which is measured by these data, including total output of poultry eggs (valuing 435200000, unit: kg), total output of aquatic products (valuing 541717000, unit: kg), total output of pork (valuing 1120717000, unit: kg), total output of poultry meats (valuing 340600000, unit: kg), their grain consumption coefficient (valuing 1.7, 1.02, 2.14, and 2.14 respectively), number of hogs (valuing 11671900) and the grain consumption per hog (valuing 0.2, unit: kg), as well as the total permanent population (valuing 31243200, unit: person).

Fourth, the value of F_4 is 0.73 kg/person, by applying the sown area of middle rice, soybean, or corn (valuing 655137, 438329, and 96921 respectively, unit: ha), the total seed volume of middle rice, soybean, or corn per sown area (valuing 8.65, 17.87, and 94.73 respectively, unit: kg/ha), and the total permanent population (valuing 31243200, unit: person), into measurement model introduced by Tang and Li [1]. The value of the total seed volume of middle rice, soybean, or corn per sown area in 2019 is calculated by trend extrapolation method through data from to 2005-2018.

Fifth, the value of F_5 is 20.65 kg/person, which is obtained by total grain yield (valuing 10752000000, unit: kg) and the loss rate during storage and transport process (valuing 0.06). Finally, $L_{P,1}$ is equal to the sum of F_1 (263.51), F_2 (11.70), F_3 (124.70), F_4 (0.73) and F_5 (20.65), valuing 421.29 kg/person, assuming $L_{P,1}$ is the same in 9 county-level administration districts, as shown in **Supplementary Table S2**.

$L_{P,2}$ is per capita freshwater consumption. The calculation for the value of $L_{P,2}$ can be obtained by the volume of freshwater consumption (W_D) dividing total number of permanent population (P_R). The results of $L_{P,2}$ is shown in **Supplementary Table S2**.

This paper refers to Chongqing Urban and Rural Master Plan During 2007-2020 and assumes that demand for land area per capita in 2019 is equal to the planning land area per capita in 2020. Therefore, the value of $L_{P,3}$, $L_{P,4}$, $L_{L,2}$, $L_{L,3}$, $L_{L,4}$, and $L_{L,5}$ is 111.87, 11.59, 10.54, 2.83, 11.95, and 10.54 respectively, unit: m^2/person, as shown in **Supplementary Table S2**.

$L_{L,1}$ is demand for residential land area per capita, which can be divided into demand for rural residential land area per capita (A_R) and demand for urban residential land area (A_U). This paper refers to Code for Classification of Urban Land Use and Planning Standards of Development Land: GB 50137-2011, the value of A_U is 29.5 (unit: m^2/person). On the other hand, demand for rural residential land area per capita is equals to rural residential land area per capita, Yuzhong, Dadukou, Jiangbei, Shapingba, Jiulongpo, Nan'an, Beibei, Yubei, and Ba'nan valuing 0, 314.68, 65.37, 302.64, 237.31, 189.04, 222.89, 127.43, and 341.48 m^2/person respectively. As A_U is more important than A_R, this paper selects the proportion of urban permanent population and the proportion of rural permanent population as the weighting value. The results of $L_{L,1}$ are shown in **Supplementary Table S2**.

$L_{E,1}$ is per capita carbon emission. To obtain the value of $L_{E,1}$, the carbon emission volume (T_C) in 2019 is calculated by trend extrapolation method through data during 1997-2017. The value of $L_{E,1}$ is equals to the ratio index of T_C and total number of permanent population (P_R), as shown in **Supplementary Table S2**.

$L_{E,2}$ is per capita waste discharge. By applying $load_{xyz}$, A_{xy}, and P_R into Equation (7), the value of $L_{E,2}$ in the 9 county-level administration districts in 2019 is calculated, as shown in **Supplementary Table S2**.

References

1. Tang, H.; Li, Z. Study on per capita grain demand based on Chinese reasonable dietary pattern. Scientia Agricultura Sinica 2012, 45, 2315-2327 (in Chinese).
2. National Land and Resources Standardization Techinical Committee. (2019). Technical regulation of the third nationawide land and resources survey: TD/T 1055-2019.
